# Are white storks addicted to junk food? Impacts of landfill use on the movement and behaviour of resident white storks (*Ciconia ciconia*) from a partially migratory population

**DOI:** 10.1186/s40462-016-0070-0

**Published:** 2016-03-15

**Authors:** Nathalie I. Gilbert, Ricardo A. Correia, João Paulo Silva, Carlos Pacheco, Inês Catry, Philip W. Atkinson, Jenny A. Gill, Aldina M. A. Franco

**Affiliations:** School of Environmental Sciences, University of East Anglia, Norwich Research Park, Norwich, NR4 7TJ UK; Centre for Ecology, Evolution and Environmental Changes (Ce3C), Departamento de Biologia Animal, Faculdade de Ciências, Universidade de Lisboa, 1749-016 Lisboa, Portugal; Centro de Ecologia Aplicada Prof. Baeta Neves and InBio, Rede de Investigacão em Biodiversidade e Biologia Evolutiva, Instituto Superior de Agronomia, Universidade de Lisboa, Tapada da Ajuda, 1349-017 Lisboa, Portugal; British Trust for Ornithology, BTO, The Nunnery, Thetford, Norfolk IP24 2PU UK; School of Biological Sciences, University of East Anglia, Norwich Research Park, Norwich, NR4 7TJ UK

**Keywords:** Movement ecology, Utilization distribution, Foraging ecology, Tracking, Birds, Foraging behaviour, Adaptation, Partial migration, Residency, Avian

## Abstract

**Background:**

The migratory patterns of animals are changing in response to global environmental change with many species forming resident populations in areas where they were once migratory. The white stork (*Ciconia ciconia*) was wholly migratory in Europe but recently guaranteed, year-round food from landfill sites has facilitated the establishment of resident populations in Iberia. In this study 17 resident white storks were fitted with GPS/GSM data loggers (including accelerometer) and tracked for 9.1 ± 3.7 months to quantify the extent and consistency of landfill attendance by individuals during the non-breeding and breeding seasons and to assess the influence of landfill use on daily distances travelled, percentage of GPS fixes spent foraging and non-landfill foraging ranges.

**Results:**

Resident white storks used landfill more during non-breeding (20.1 % ± 2.3 of foraging GPS fixes) than during breeding (14.9 % ± 2.2). Landfill attendance declined with increasing distance between nest and landfill in both seasons. During non-breeding a large percentage of GPS fixes occurred on the nest throughout the day (27 % ± 3.0 of fixes) in the majority of tagged storks. This study provides first confirmation of year-round nest use by resident white storks. The percentage of GPS fixes on the nest was not influenced by the distance between nest and the landfill site. Storks travelled up to 48.2 km to visit landfills during non-breeding and a maximum of 28.1 km during breeding, notably further than previous estimates. Storks nesting close to landfill sites used landfill more and had smaller foraging ranges in non-landfill habitat indicating higher reliance on landfill. The majority of non-landfill foraging occurred around the nest and long distance trips were made specifically to visit landfill.

**Conclusions:**

The continuous availability of food resources on landfill has facilitated year-round nest use in white storks and is influencing their home ranges and movement behaviour. White storks rely on landfill sites for foraging especially during the non-breeding season when other food resources are scarcer and this artificial food supplementation probably facilitated the establishment of resident populations. The closure of landfills, as required by EU Landfill Directives, will likely cause dramatic impacts on white stork populations.

## Background

The migratory patterns of animals are changing in response to global environmental change [1–3]. Many previously wholly migratory bird species that used to winter in sub-Saharan Africa are forming resident populations in their southern European breeding grounds [4, 5]. The migratory strategy an individual adopts may impact on subsequent survival and lead to different population dynamics between migrant and resident individuals. Even small differences in survival and productivity associated with migratory strategy may lead to very rapid changes in the proportion of the overall population that migrates. Individuals face a cost-benefit trade off concerning whether to stay or migrate [6]. Whilst migrants undergo energy demanding large-scale movements, residents are able to occupy the best breeding areas. Resident birds are known to breed earlier than migrants, have larger clutches [7] and early nests are known to have higher breeding success [8–10]. However, residents usually experience less favourable environmental conditions in the breeding areas during the winter, affecting their survival directly or indirectly through food availability [11, 12]. The ability of resident birds to find food resources during this period may therefore be key for their survival. The ecology of migratory species that have become resident is not well understood. In particular, the non-breeding season movement behaviour of migratory populations that have recently become sedentary is poorly studied. Understanding the role of food availability in driving changes in resident bird distribution and movement behaviour will improve our ability to predict how partially migratory species may respond to future climate and environmental change and assist in designing effective conservation strategies.

Food supplementation in birds has been shown to advance bird phenology [13], affect singing behaviour [14], increase fledging success [15] and impact individual fitness and survival [16]. Whilst birds are known to congregate and preferentially nest close to reliable artificial food resources [17], there is relatively little knowledge on the impacts of their utilization on daily movement patterns and migratory behaviour.

Artificial food available from landfill sites may have facilitated the recent establishment (since the 1980s) of resident white storks populations in Iberia [18]. This is within the lifetimes of individual birds in this long-lived (up to 25 years in the wild), iconic species. The causes of these changes in behaviour are not fully established but milder European winter temperatures due to climatic change [7], increased winter food availability from landfill sites [19] and foraging on the invasive Red Swamp Crayfish (*Procambarus clarkii*) in rice fields [20] have been proposed as likely factors. Foraging on landfill is undoubtedly a major influence as 80 % of overwintering white storks in Iberia congregate near landfill sites [21] and landfill forms 68.8 % of local diets in both adults and juveniles throughout the year [22]. White storks also preferentially nest near landfill sites [21] which has consequences for population distribution and range expansion patterns. Foraging on landfill is also a relatively new occurrence in the Eastern Europe stork populations [23]).

The number of overwintering white storks in Portugal has increased dramatically in recent decades (from 1,187 individuals in 1995 to 10,020 in 2008 [24] and to approximately 14,000 birds in 2014 (Rosa, personal communication)), simultaneously, the number of migrant individuals crossing the Straits of Gibraltar has increased by 86.4 % between 1985 and 2004 [25] and recent data indicates this trend continues [26] suggesting that the overall population is increasing, not simply changing in migratory behaviour.

This study is the first to assess the consequences of reliable and abundant food resources (landfill sites) on the large-scale movement patterns of a recently established resident population of a previously wholly migratory species. We assess the spatial and temporal changes in movement behaviour throughout the year using newly developed GPS/GSM technology. We quantify the extent and consistency of landfill use by resident individuals during the breeding and non-breeding seasons and assess its influence on nest use, daily travel, foraging and non-landfill foraging ranges.

## Methods

### Study area and study system

Data loggers were deployed on 48 birds captured on active landfill sites during the winters of 2012/13 (*n = 15*) and 2013/14 (*n = 33*). Licenses to catch and deploy loggers were granted by the Instituto da Conservação da Natureza e das Florestas (ICNF). Five landfill sites across south-central Portugal were used: Aterro Sanitário de Ermidas do Sado (38.021444, −8.353319, *n = 11*), Aterro Sanitário de Vila Ruiva (38.243040, −7.952321, *n = 10*), Aterro Sanitário Intermunicipal de Évora (38.538004, −7.971274, *n = 16*), Aterro Sanitário da Herdade do Montinho, Beja (37.924916, −7.864950, *n = 8*) and Aterro Sanitário do Barlavento, Portimão (37.214041, −8.522350, *n = 3*). Birds nested a maximum of 48.2Km from their capture location. The surrounding habitat was largely Mediterranean cork oak woodland (*montado*), a traditional low intensity management system consisting of savannah-like grassland with Cork oak (*Quercus suber*) and holm oak (*Quercus rotundifolia*) trees in varying densities used for cattle grazing and low intensity agriculture. The surrounding area also included non-irrigated agriculture, often in multi-annual crop rotation cycles, irrigated agriculture, rice fields and small plantations of olive trees and of deciduous and evergreen forestry. Urban settlements were mostly low density, apart from the city of Évora (population 56,600).

Storks were captured using nylon and rubber leg lassos and a remotely activated, baited clap net. Both were deployed on the actively worked landfill and monitored continuously. Birds were detained for maximum of half an hour after capture. They were colour ringed on each leg and sex was estimated at time of capture from physical characteristics (body size, ruff size and bill length), a method known to be correct in 89 % of cases [27]). In this study, sex was subsequently confirmed as correct in all six birds who were observed copulating.

There appeared to be no adverse effects of the deployment process. Several individuals were resighted in the days immediately following logger deployment and were behaving normally. Capture dates, total tracking time and the number of days of data available for each season are listed in Table 1.

**Table 1:** Sex, start and end dates and number of days of data for each white stork

Sex^a^	Capture Date	End of Breeding	No. Days of Data		Total	Breeding Onset Verification	Date Last Tracked
			Non-breeding	Breeding			
(M)	15/11/2012	06/06/2013	118	85	203	A + F	14/02/2014
(F)	15/11/2012	28/05/2013	123	71	194	A	13/05/2014
F	16/11/2012	21/06/2013	112	105	217	A + F	16/09/2013
(F)	24/11/2012	04/06/2013	119	73	192	A	11/01/2014
(M)	24/11/2012	30/03/2013	126	62	188	A + F	05/06/2013
(F)	25/11/2012	17/04/2013	101	42	143	A	21/07/2013
(M)	25/11/2012	19/05/2013	113	62	175	A + F	24/06/2013
(F)	30/12/2012	08/06/2013	78	82	160	A + F	24/09/2013
(F)	30/12/2012	02/05/2012	99	24	123	A	15/09/2013
(M)	17/01/2013	31/05/2013	66	68	134	A	12/09/2013
(F)	17/01/2013	12/06/2013	68	78	146	A	12/06/2013
F	29/11/2013	06/06/2014	90	99	189	A + F	10/10/2014
F	08/12/2013	10/06/2014	88	96	184	A + F	11/11/2014
(M)	11/12/2013	01/06/2014	82	90	172	A	28/06/2014
(M)	11/12/2013	01/06/2014	83	89	172	A + F	26/06/2014
(M)	15/01/2014	01/06/2014	83	89	137	A	21/08/2014
(F)	02/02/2014	17/04/2014	50	24	74	A + F	01/06/2014

Breeding onset from field observations (F) and/or accelerometer data (A)

^a^Sex: in brackets was estimated at time of capture from physical characteristics, without brackets was confirmed from copulation

Nests of tagged birds were easily detected as a location of tightly clustered GPS coordinates in continuous use. These locations were visited to confirm nest occupancy. All tagged bird nests were visited on 2–3 occasions throughout the breeding season (March and late May/early June) to monitor breeding parameters. Nests were observed with a telescope and, where possible, a camera pole was used to look in to the nest on each visit to assess clutch size and chick age (based on visual assessment of bill length and plumage development).

### Data loggers and the identification of behaviours

Newly developed GPS-ACC data loggers, developed by our team, were used in this study. All loggers were back-mounted on a teflon harness with biodegradable stitching to prevent lifelong placement. Loggers weighed 90 g (battery powered) and 45 g (solar powered), less than 4 % of the total mass of the bird. After deployment, loggers quickly sank below the feathers minimising drag.

Loggers were programmed to transmit 5 times per day at 5 am, 8 am, 11 am, 2 pm and 5 pm GMT. Each data burst obtained 10–20 consecutive GPS fixes (±20 m accuracy) and 3D accelerometer readings (at 1Hz with a sensitivity of ±6 G), once per second. Data are automatically transmitted via GPRS using the GSM mobile phone network every 2 days to a web platform.

**Fig. 1:**
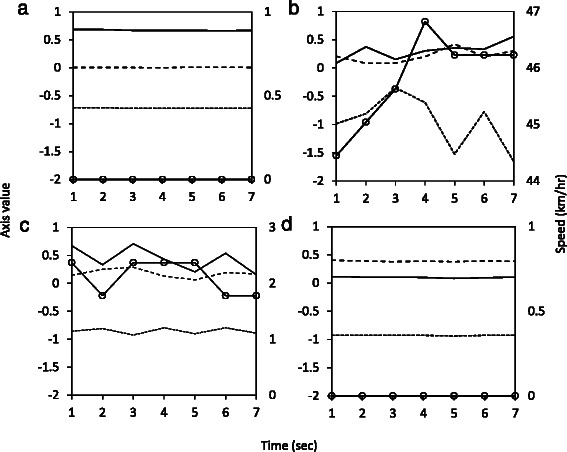
White stork behaviours identified using the accelerometer data: **a** inactive (standing), **b** flight, **c** foraging, **d** tending eggs. The three axes and speed are represented, X axis- surge (black line), y axis -sway (dashed line), z axis -heave (dotted line) and speed (line with circles)

The information obtained from the accelerometer axes X (surge), Y (sway) and Z (heave in gravity) plus speed (derived from GPS positions) were used to determine behaviour during each data burst. Assigning a behaviour to each GPS fix allowed non-foraging behaviour (flight, inactive) to be excluded from foraging analysis, it was not to derive time budgets. The information for each variable was summarized by calculating the mean, standard deviation, max, min and range. Subsequently, each data burst was classified into four behaviour categories: inactive (standing and/or preening), foraging, flight and tending eggs. In the rare occasion that multiple behaviours were captured in a single data burst, the behaviour occurring at the end of the data burst was used. This was because GPS position was fixed most accurately later in the transmission. Initially, the behaviours in a set of 500 randomly selected data bursts were manually classified for 6 birds based on field observations and by analysing accelerometer output simultaneously with video footage of the logger on the birds back. Reconstructions of accelerometer output in real-time with a hand held accelerometer linked to a computer were also used to validate the behaviours. We used a 75–25 % split to separate these behaviours into two independent sets: 375 randomly selected behaviours were used for training and 125 for validation of a classification tree model of the four behaviour classes. Behaviour classification analysis was done with R using the *rpart* library and the final classification-tree model was selected based on the lowest training data cross-validation error after 10 runs. The overall model accuracy was assessed using a multi-class AUC test (HandTill 2001, library [28]), resulting in a single AUC value of 0.97, indicating a good level of classification of the four behaviours. Example graphs of each behaviour are shown in Fig. 1. Individual classification performance can be found in the confusion matrix (Appendix 1).

### Determination of onset and end of breeding

For the purpose of this study, the non-breeding season is defined as the period between capture (November–February) and initiation of breeding detected in the tri-axial accelerometer data and verified with field observations.

The accelerometer data enabled the identification of the initiation of breeding designated *“*tending eggs*”,* characterised by birds looking down into the nest from a standing position. Our model predicted tending eggs behaviour with 87.0 % accuracy (Appendix 1). This characteristic body position is observed in the field during the early breeding season and is a good indication that eggs are present. Tending eggs only occurred for a period of between 4 – 5 weeks during the incubation phase, only occurred in GPS fixes on the nest, and was detected in all breeding birds. The onset of this behaviour in the accelerometer data is abrupt and distinctive and matched observed timings for egg laying in all tagged birds with confirmed field data (n = 9). The error between the appearance of the tending eggs behaviour in the accelerometry data and field observations of the presence of eggs was ±1 day. For the remaining birds (n = 8), egg lay date is based on accelerometry and hatch date was also estimated based on chick ages observed by telescope (Table 1).

The breeding season is defined as the period from the initiation of breeding until either (1) breeding failure (n = 6), (2) chicks fledged (n = 8) or (3) the logger stopped transmitting (n = 3). Failed breeders abruptly abandoned the nest for a period of 3–5 days. Fledge date was known precisely in 5 successful nests because chicks were tagged with GPS devices (n = 2 nests) or chicks were seen fledging (n = 3 nests). The remaining 3 nests were monitored and fledge date was estimated from chick age and development during late season colony visits. After fledgling chicks usually remain in the vicinity of the nest and continue to be supplementary fed by the adults for a period of one or two weeks, so for all nests fledgling date was defined as the earliest possible fledge date.

This analysis focuses exclusively on the behaviour of breeding birds during the pre-breeding and breeding period, no post breeding data are included. Similarly, juveniles (n = 3) and non-breeding birds (n = 5) were also omitted. Juveniles were distinctive due to their dispersive nature and lack of obvious cluster of GPS fixes which would have marked the location of a nest. Other non-breeding birds were associated with a nest but displayed no breeding behaviour as determined from accelerometer data (no tending eggs behaviour) and field observations.

One tagged individual died prior to breeding. Twenty two loggers stopped transmitting prior to or shortly into the breeding season and therefore could only be included for consideration of maximum distance travelled between nest and landfill. Here we present results from 17 tagged breeding birds, *n = 7* assessed to be male and *n = 10* assessed to be female (Table 1).

### Non-breeding and breeding season landfill use

GPS fixes from all data bursts (excluding fixes where the bird was in flight), were used to ascertain the percentage of fixes occurring on landfill, non-landfill habitat and within 20 m of the nest. A 50 m buffer was drawn around each landfill and all points within this buffer were considered as landfill. This 50 m buffer captured occasions where birds were disturbed and temporarily flushed off the landfill to just beyond the site perimeter. Distance from the nest to landfill was determined using the minimum straight-line distance between the nest and the centre of the utilised landfill site. GPS fixes within 20 m of the nest were removed from foraging analyses and analysis of seasonal reliance on landfill because on the nest the birds are usually inactive. Comparisons of percentage landfill use during breeding and non-breeding season (Figs. 3 and 4a and b) use only fixes outside the nest to reduce bias resulting from increased nest use during the breeding season.

### Daily distance

Non-breeding and breeding season mean daily distance travelled was calculated using GPS locations only from days where all 5 data bursts were available (including fixes in flight). This varied between individuals from 34.2 to 98.8 % of total data bursts (mean ± SE non-breeding: 77.6 ± 3.7, breeding: 68.2 ± 4.9). The distance between successive GPS positions (pairs of latitudes and longitudes) were calculated in kilometres then summed to obtain a daily totals. Daily totals were then used to create a non-breeding and breeding season mean for each individual.

### Non-landfill foraging range

Foraging range was derived by calculating the 50 and 95 % utilization distribution kernels for each bird using only data bursts where accelerometer information indicated the bird was foraging. Kernel polygons were determined with the R library *ade-habitat* and imported into ArcGIS to calculate kernel area. Data bursts where the individual was standing, flying or engaged in breeding behaviour were excluded. The aim of this analysis was to investigate natural foraging habits so data bursts occurring within 50 m of landfill sites were also removed.

### Statistical analysis

Data are normally distributed so paired *t*-tests were used to assess seasonal differences in the percentage of GPS fixes spent by the nest, on landfill and in non-landfill habitat. Linear regressions were used to explore the influence of distance from nest to land fill site on seasonal nest use and landfill use in the breeding and non-breeding seasons and to determine the relationship between nest-landfill distance and the percentage of data bursts assigned as foraging and resting behaviour. Paired *t*-tests and non-linear regression were used to compare differences in mean travel distances between seasons. Foraging range size (50 and 95 % kernels) were log transformed for normality and linear-regression was used to test the relationship between range size with nest-landfill distance.

## Results and discussion

### Results

Forty-eight birds were tracked for a total of 155 months (mean per individual 9.1 ± 3.7 months, mean fixes per day: 4.17 +/− 0.15). This study focuses on 10,425 data bursts (613.2 ± 41.3) from 17 birds, 5758 during the non-breeding season (338.7 ± 26.6) and 4667 during breeding (274.5 ± 23.2).

### Seasonal foraging habitat, landfill and nest use

**Fig. 2:**
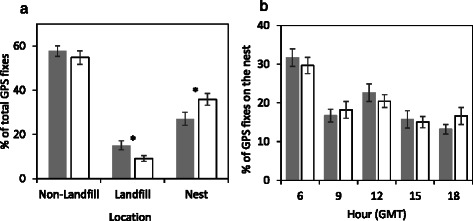
White stork habitat and nest use during the non-breeding (filled bars) and breeding (open bars) seasons. **a** Seasonal differences in percentage of total GPS fixes (±SE, excluding flight) registered in non-landfill habitat (t(16) = 1.465, *p* = 0.162), landfill sites (t(16) = 2.63, *p* = 0.018), and on the nest (t(16) = −4.36, *p* = 0.001). **b** Frequency of GPS fixes occurring on the nest during each of the 5 daily data bursts as a percentage (±SE) of all transmissions within 20 m of the nest. Asterisks indicate statistically significant differences

The percentage of total GPS fixes (excluding flight) on non-landfill habitat was similar in the breeding and non-breeding seasons (Fig. 2a, paired *t*-test, t(16) = 1.465, *p* = 0.162). During the non-breeding season, a large percentage of total GPS fixes (excluding flight) were spent on the nest (mean 27.1 % ± 2.97) with 25 % of the birds (assessed as both males and females) spending up to 49.7 % of GPS fixes within 20 m of the nest. Individuals of both sexes were found on their nests throughout the day and there was no significant difference in the hour of nest attendance between seasons (Fig. 2b), 22.6 % ± 2.24 of non-breeding GPS fixes on the nest occurring at midday (Fig. 2b). Time spent in the nest was significantly higher during breeding season (Z = −2.956, *p* = 0.003). The percentage of GPS fixes on the nest was not related to the distance between nest and landfill site during either the breeding F(1,15) = 0.011, *p* = 0.915) or non-breeding F(1,15) = 0.035, p = 0.855) seasons. Behavioural data, derived from accelerometery, indicated that in the majority of fixes on the nest the birds are inactive (inactive fixes within 20 m of the nest: 87.2 % ± 6.4 non-breeding, 86.9 % ± 6.4 breeding). Post breeding data from 8 individuals tracked until at least September (Table 1) indicated that all birds continued to remain on their nests after chicks fledged.

**Fig. 3:**
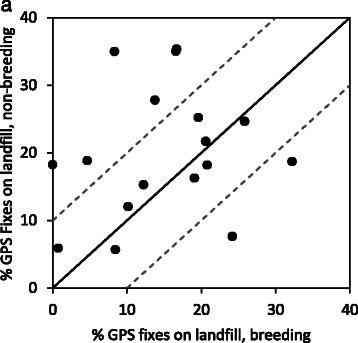
Frequency of GPS fixes away from the nest (excluding flight) occurring on landfill sites in the breeding and non-breeding seasons for 17 white storks. Dashed lines are the 10 % intervals around the line that represents equal use of landfill in both the non-breeding and breeding seasons. 6 birds use landfill less in the breeding season (points above dashed line), 9 individuals use landfill equally in both seasons (points inside dashed lines), and 2 birds use landfill more in the breeding season (points below dashed lines)

All 17 individuals that were tracked during non-breeding and breeding seasons used landfill to some extent in the non-breeding period, whilst one individual did not use landfill at all during breeding and a second only rarely (0.7 % of data bursts, Fig. 3). Although there were individual differences, overall percentage of fixes on landfill (excluding nest fixes) was higher during the non-breeding season (mean ± SE 20.1 % ± 2.3 of GPS fixes) compared to the breeding season (14.9 % ± 2.2, paired *t*-test, t(16) = 2.63, *p* = 0.018). 35.3 % (*n = 6*) of individuals had higher attendance on landfill during the non-breeding season, the majority of individuals 52.9 % (n = 9) used landfill approximately equally in both seasons and two birds used landfill more during breeding (Fig. 3).

### Impact of distance between nest and landfill on landfill use

**Fig. 4:**
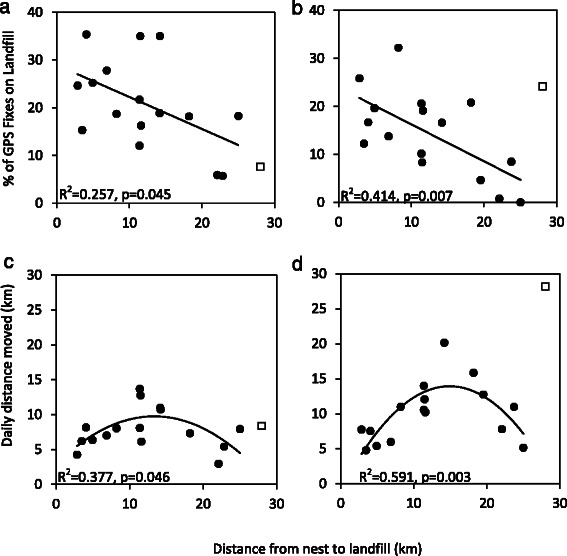
Percentage of GPS fixes on landfill (excluding fixes in flight and within 20 m of the nest) in relation to distance from nest to the landfill site during **a** non-breeding and **b** breeding seasons. Total daily distance moved (derived from all available fixes, including flight and nest) in relation to distance between the nest and the landfill site during **c** non-breeding and **b** the breeding season. One individual was considered an outlier (unfilled square) and was excluded from the linear regressions, see results section

Regardless of differences in landfill use between seasons, individuals closer to landfill used this resource more frequently than their more distant conspecifics and landfill attendance declined with increasing straight-line distance between the nest and landfill site in both seasons (Fig. 4, non-breeding R^2^ = 0.257, p = 0.045, breeding: R^2^ = 0.414, *p* = 0.007). Distance from nest to landfill is strongly correlated with frequency of landfill use during breeding (Fig. 4b).

One white stork had a different strategy compared to all other birds in this study (unfilled square symbol, Figs. 4 and 6). This individual was frequently detected on landfill during breeding, despite having the largest nest-landfill distance (28.1 km). This was probably due to a lack of non-landfill resources in the vicinity of the nest during breeding, likely associated with the local timing of rice field drainage. This bird was considered an outlier, and was not included in the analyses.

White storks travelled greater maximum distances during the non-breeding season to visit landfill sites (t(16) = −2.280, *p* = 0.038). During the non-breeding season almost one quarter (23.5 %) of tagged birds travelled over 25 km to reach landfill while in the breeding season the maximum distance travelled was 28.1 km. When considering days with all 5 daily GPS fixes available (including flight), we found a positive quadratic response, particularly during breeding (Fig. 4d). The mean distance travelled increased with distance to the nest until approximately 14–15 km and then as the nest-landfill distance increased further, the mean distance travelled decreased. This suggests a threshold distance that birds will preferentially travel to landfill. Birds with nests located at this distance travelled larger daily distances (travelling further to visit landfill sites) than birds close to landfill or further away.

Birds exhibited higher daily distance displacement during the breeding season (mean ± SE 11.19 km ± 1.46 per day) compared with the non-breeding season (7.91 km ± 0.69, paired t(16) = −2.37, *p* = 0.031).

### Foraging behaviour and foraging range

**Fig. 5:**
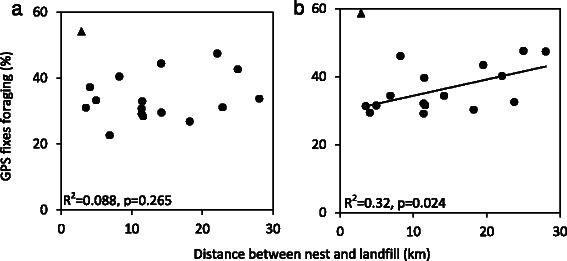
Percentage of foraging behaviour (defined from all GPS fixes) in relation to distance from nest to landfill during the **a** non-breeding and **b** breeding seasons. One individual (triangle) had an exceptionally high percentage of foraging behaviour GPS fixes during both the non-breeding and breeding seasons and was excluded from the linear regressions presented in this figure. Analysis including this individual showed no significant relationship

During the breeding season, birds nesting further from landfill had a higher percentage of total GPS fixes (excluding flight) associated with foraging behaviour (Fig. 5b). While during the non-breeding season there was no effect of distance to landfill on the percentage of foraging GPS fixes (Fig. 5a). All birds, except one, marked as a triangle, fitted this pattern. Analysis including this individual found no relationship in either the non-breeding (R^2^ = 0.001, *p* = 0.919, mean Mahalanobis distance ± SD: 0.941 ± 1.017) or breeding season (R^2^ = 0.037, *p* = 0.461, Mahal: 0.941 ± 0.913).

Additionally, excluding only GPS fixes in flight, birds that use landfill in higher percentages also had higher percentages of standing (resting) behaviours during the breeding season only (breeding: R^2^ = 0.804, *p* = 0.000, non-breeding: R^2^ = 0.009, *p* = 0.724).

**Fig. 6:**
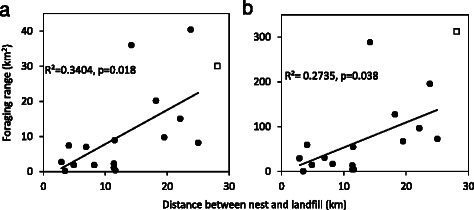
**a** 50 % and **b** 95 % UD Kernels constructed from GPS fixes associated only with data bursts showing foraging behaviour. One individual (unfilled square) is excluded from the linear regressions (see results section)

During the breeding season, white storks increased their non-landfill foraging range (measured by kernel 50 and 95 % generated using all GPS fixes associated with foraging behaviour) with increasing distance between the nest and the landfill site (50 % kernel: F(1,14) = 7.225, R^2^ = 0.340, *p* = 0.018, 95 % Kernel: F(1,14) = 5.270, *p* = 0.38, R^2^ = 0.273, Fig. 6). There was no significant increase in foraging range with distance between nest and landfill during non-breeding (50 % kernel: (F(1,14) = 0.19, R^2^ = 0.013, *p* = 0.67, 95 % Kernel: F(1,14) = 0.130, R^2^ = 0.009, *p* = 0.72).

## Discussion

### Winter nest use

This study provides first confirmation of year round nest use, an entirely new behaviour that has developed as the Iberian population of white storks shifted from being wholly migratory to partially migrant. There is no evidence from previous monitoring studies [7, 29, 30] to suggest ringed storks occupied their nests all year, perhaps because year-round nest use is a recent phenomenon. Data from tracking studies of migratory white storks indicate that, whilst highly faithful to their breeding grounds, individuals have little wintering site fidelity and pairs do not winter together [31]. Landfill sites provide abundant food resources that are reliable in both space and time, thus likely contributing to enabling individuals to remain in their breeding territory and on their nests year-round. This is extremely rare in temperate zones because, during winter, resident individuals of other species usually perform regional or local movements away from their breeding territory and/or form loose flocks that are highly mobile to track limited, dynamic winter food resources [11].

Nest use and maintenance was observed throughout the day during the non-breeding season (Fig. 2b) with both males and females spending up to 49.7 % of GPS fixes within 20 m of the nest. This suggests the nest is defended during the winter rather than simply being used as a roost site at night. Field observations confirmed it is not uncommon to see pairs on the nest throughout the day engaged in nest defending and repair (Gilbert, personal observations) during the non-breeding season. Whilst in the vicinity of the nest birds are inactive (standing/preening) rather than engaged in foraging behaviour, which is a significant time investment during winter when daylight foraging hours are shorter.

Nests near guaranteed food supply from landfill are highly desirable locations [21, 32] and therefore it was predicted these would require more defending than nests in non-landfill locations. However, the lack of correlation between the percentage of GPS fixes on the nest and either distance between nest and landfill or frequency of landfill attendance indicates individuals defend their nest regardless of proximity to landfill. This may be partially driven by other factors including proximity to high quality non-landfill habitat, colony size and the high white stork population density found in Iberia. It is unclear if white storks are limited by the availability of suitable nest locations around landfill sites. White storks nest in close proximity to each other on myriad structures from trees to pylons and other man-made constructs which suggests many nest options, [32] however nest sites within specific colonies may be limited [33].

Residency enables an advance in breeding phenology and can increase the breeding success of residents compared to returning migrants in the same population. This may be because residency enables occupation of the most favorable nesting locations [34] and, in many species, facilitates earlier laying date [7, 10]. Nests near landfill fledge significantly more chicks [21] and fledging success has been demonstrated to decline by 8 % per kilometre distance from the feeding location [15]. In populations of white stork that do not use landfill, arrival date is strongly correlated with fledgling success due to the seasonal decline in food availability [35, 36]. Abundant food from landfill sites therefore mitigates the seasonal decline in food availability.

### Impact of nest distance on seasonal reliance on landfill

Overall, white storks were more reliant on landfill during the non-breeding season (Figs. 2 and 3). All individuals in this analysis were caught on landfill sites so it was expected that all used landfill to some extent, however in the breeding season two birds (11 %) did not use landfill. This study shows landfill site use varies considerably and is lower in Iberia than previously described [22]. Individuals nesting closer to landfill utilized this resource more frequently in both seasons than those nesting further away (Fig. 4a and b) and landfill use declined with increasing distance between the nest and landfill, even during the non-breeding season, indicating that in both seasons distance from nest to landfill is the dominant factor determining reliance on landfill. During chick rearing, due to energy requirements and travelling time constraints, this relationship was expected. However, it was surprising during the non-breeding season, and may be because resident storks now also occupy their nests during the non-breeding season, rather than forming loose roaming winter flocks, thus foraging occurs from a central point, the nest, throughout the year.

The lower frequency of use of landfill during breeding is possibly due to prey size. Adult white storks may prefer to feed smaller food items foraged in non-landfill habitats to their chicks. White storks supplementary fed with large items (rats, small chickens, fish) had similar foraging rates to nests that were not supplementary fed until chicks were over 20 days old and able to handle larger items [37]. This is consistent with similar behaviour showed by gulls. Herring gulls (*Larus argentatus*) preferred soft, small foods (e.g. earthworms) in the first days after chick hatching; but immediately switched back to the more energetically profitable strategy of foraging on landfill as soon as chicks could swallow larger items [38]. Similarly, Yellow legged gulls (*Larus michahellis*) were observed to shift their diets from landfill to other terrestrial habitats when feeding feed chicks [39]. It is also possible that non-landfill food resources are more abundant during breeding and this may contribute to decreased landfill use during this season.

During the non-breeding season white storks travel larger distances to visit landfill sites. One in every four breeding birds analysed travelled over 25 km and one bird travelled 48.2 km from its nest to the landfill during non-breeding, while in the breeding season the maximum distance travelled was 28.1 km. This revises previous work that suggest Iberian white storks travel 12 km to reach landfill [21]. Massemin-Challet et al*.* [7] defined non-landfill colonies as ones 15 km from landfill and Moritzi et al*.* [37] suggested storks travel an additional 4 km to reach supplemental food, both of which are under estimates for Iberian storks.

Distance from nest to landfill defines how far an individual is prepared to travel each day as well as how heavily landfill is used. The relationship is non-linear so daily distance moved increased with distance from the landfill whilst it remained beneficial (both energetically and in terms of leaving the nest undefended) to visit landfill (Fig. 4c and d). Thus, individuals who nest close to landfill use landfill more and travel lower daily distances. This effect is particularly strong during breeding when birds travel greater daily distances and nest affinity is stronger.

### Foraging behaviour and foraging range

During non-breeding there was no significant effect of nest-landfill distance on the percentage of GPS fixes spent foraging (Fig. 5a) or resting. This may be associated with seasonal changes in the quality of non-landfill habitat surrounding the nest, in particular the abundance of red swamp crayfish. This important prey species is now prevalent in water ways across Iberia, particularly rice fields, and is more accessible to storks during winter when water levels are high [40]. Crayfish abundance in the vicinity of the nest may therefore render the correlation between nest-landfill distance and percentage of GPS fixes spent in foraging activities less significant. During breeding, there is a general trend for birds close to landfill to have fewer foraging behaviour GPS fixes than those nesting further away (Fig. 5b). This may be because energetic requirements during chick provisioning are being more rapidly met in nests close to landfill compared to nests further away. The individual that did not follow this trend foraged extensively in both seasons, despite proximity to landfill (Fig. 5), and is suspected to be a young, inexperienced bird. Inclusion of this individual removed the significance of the relationship between foraging behaviour and nest-landfill distance, suggesting a greater sample size is required in order to fully capture the range of behavioural responses. During breeding the majority of foraging occurs close to the nest so individuals nesting close to landfill are more likely to visit landfill and the average distance at which it compensates to visit landfill decreases.

Individuals nesting close to landfill had higher percentages of resting behaviour GPS fixes during the breeding season than those further away. This may indicate another possible benefit of being close to landfill that could have important fitness consequences and should be investigated further by future studies.

**Fig. 7:**
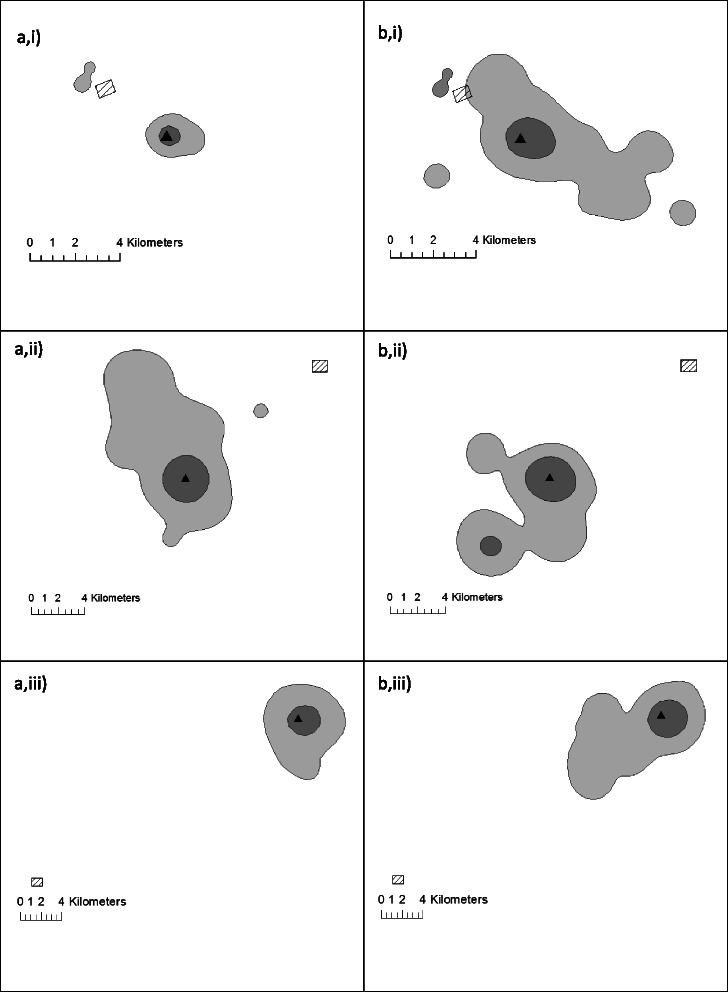
Typical non-landfill foraging ranges for 3 individuals (i,ii,iii) nesting at varying distances from landfill. The non-breeding season (**a**) and the breeding season (**b**) are depicted. 50 % (dark grey) and 90 % (light grey) UD Kernels from foraging GPS fixes in non-landfill habitat. Nests (black triangles) were located at 2.9 km (i), 11.5 km (ii) and 25.0 km (iii) from landfill. The shaded rectangle indicates the position of the landfill

Foraging range in non-landfill habitat increased with distance from the nest to landfill site indicating that birds nesting further from landfill forage primarily in non-landfill habitat and require larger foraging areas. This was only significant during the breeding season (Fig. 6). Kernel analysis indicated that across individuals and seasons, at least 50 % of non-landfill foraging occurred immediately around the nest (Fig. 7), which is congruent with findings of previous studies [41–43]. Landfill visits were usually specific, long distance excursions away from the nest that rarely included stops in non-landfill habitat *en route*. This may explain why distance from landfill had no effect on non-landfill foraging area during the non-breeding season. It also highlights the possibility for year-round depletion of local resources surrounding the nest, particularly in the non-breeding season when non-landfill resources may be less abundant.

The European Union Landfill Directive (1993/31/EC) set targets to progressively reduce the volume of biodegradable municipal waste entering landfills through to 2016 [44]. As a result, open-air landfills are being replaced by covered waste processing facilities that are inaccessible to birds. In the immediate future there will be a sharp reduction in the availability of food waste that will have important consequences for Iberian white storks. This study is particularly relevant as it quantifies the extent to which resident Iberian white stork population rely on artificial food prior to the closure of landfill sites.

## Conclusions

This study shows the effect of recent anthropogenic changes on the movement ecology and behaviour of a long-lived species through the provision of abundant and spatially stable food resources. This study presents robust evidence that resident white storks defend their nests year round and consequently spend a large percentage of GPS fixes attending the nest during the non-breeding season. The food resources, obtained on landfill sites, likely facilitated the establishment of resident individuals in a previously wholly migratory species. Frequency of landfill use by white storks decreases with increasing distance between the nest and the landfill, during both non-breeding and breeding seasons. During breeding birds nesting further from landfill spend proportionally more GPS fixes engaged in foraging behaviour and have larger foraging ranges in non-landfill habitat than birds nesting close to landfill sites. This will likely impact breeding success and population demography.

## Availability of supporting data

Not yet available.

## Appendix

**Table 2:** Confusion matrix

		Behaviours			
		Standing/Preening	Foraging	Flight	Tending Eggs
Predicted	1	**379**	16	1	10
	2	15	**254**	1	0
	3	0	1	**29**	0
	4	7	0	0	**37**

Performance of the decision tree model used to predict four behaviour classes: 1) standing/preening, 2) foraging, 3) flight and 4) tending eggs. True positives are in bold

## Abbreviations

GPRS: general packet radio service
GPS: global positioning system
GSM: global system for mobile communications
